# Mapping Data to Concepts: Enhancing Quantum Neural Network Transparency with Concept-Driven Quantum Neural Networks

**DOI:** 10.3390/e26110902

**Published:** 2024-10-24

**Authors:** Jinkai Tian, Wenjing Yang

**Affiliations:** 1 Intelligent Game and Decision Lab, Beijing 100071, China; 2 Department of Intelligent Data Science, College of Computer Science and Technology, National University of Defense Technology, Changsha 410073, China

**Keywords:** quantum artifical intelligence, quantum neural networks, explainable artificial intelligence, autoencoder, concept-driven

## Abstract

We introduce the concept-driven quantum neural network (CD-QNN), an innovative architecture designed to enhance the interpretability of quantum neural networks (QNNs). CD-QNN merges the representational capabilities of QNNs with the transparency of self-explanatory models by mapping input data into a human-understandable concept space and making decisions based on these concepts. The algorithmic design of CD-QNN is comprehensively analyzed, detailing the roles of the concept generator, feature extractor, and feature integrator in improving and balancing model expressivity and interpretability. Experimental results demonstrate that CD-QNN maintains high predictive accuracy while offering clear and meaningful explanations of its decision-making process. This paradigm shift in QNN design underscores the growing importance of interpretability in quantum artificial intelligence, positioning CD-QNN and its derivative technologies as pivotal in advancing reliable and interpretable quantum intelligent systems for future research and applications.

## 1. Introduction

The field of quantum computing has garnered significant attention due to its potential to solve complex problems more efficiently than classical computing [1,2,3,4,5]. In particular, quantum computing offers promising advantages in processing large-scale remote sensing data, which is critical in applications like earth observation and remote sensing [6,7,8,9]. Quantum neural networks (QNNs), leveraging the principles of quantum mechanics, have shown substantial promise in enhancing computational capabilities and addressing problems that are intractable for classical neural networks [10,11,12,13]. Foundational works, such as the Quantum Approximate Optimization Algorithm [14] and the Variational Quantum Eigensolver [15], have paved the way for integrating quantum computing with neural network architectures. Significant advancements in this domain include the introduction of quantum perceptrons [16], quantum support vector machines [5], quantum convolutional neural networks [17], quantum generative adversarial networks [18], and quantum autoencoders [19].

While these investigations elucidate the potential of QNNs, they concurrently highlight a critical challenge: interpretability. In numerous vital domains, including healthcare, finance, and remote sensing, understanding the reasoning behind a model’s decisions is as crucial as the decisions themselves. This necessity underscores the importance of model transparency and reliability [20,21,22,23,24,25,26,27]. Quantum eXplainable Artificial Intelligence (QXAI) seeks to enhance the interpretability of quantum models, thereby making them more transparent and reliable for practical applications.

Generally, the complexity of a model is directly related to its accuracy but inversely related to its interpretability. Models with simpler structures are more interpretable but tend to exhibit lower accuracy. Conversely, models with complex structures demonstrate high accuracy; however, due to the large number of parameters, complex mechanisms, and low transparency, their interpretability is relatively poor.

In practical learning tasks, a decision must be made between selecting a simple, easily interpretable model and training it, or training an optimal, complex model and subsequently developing interpretability techniques to explain it. Based on these two approaches, the interpretability of machine learning models can generally be divided into two categories: ante hoc methods [28,29,30] and post hoc methods [31,32,33]. *Ante hoc methods* refer to models that are inherently interpretable, either by training models with simple structures or by incorporating interpretability into specific model architectures, thus making the model itself interpretable. Examples of self-explainable models include linear models, generalized linear models [34], decision trees [35,36], and random forests [37]. In contrast, *post hoc methods* treat trained models as black boxes, employing developed interpretability techniques to explain them.

Despite significant advancements in the field of eXplainable Artificial Intelligence (XAI) [23,25,38,39,40,41], the development of Quantum eXplainable Artificial Intelligence (QXAI) has been comparatively gradual. Recent research on QXAI has primarily focused on post hoc methodologies [42,43,44,45,46], which are often criticized for potentially providing unreliable or misleading explanations. In contrast, inherently interpretable models offer explanations that are intrinsically aligned with the model’s computational processes. Interpretability is domain-specific and frequently constrained by structural knowledge, such as monotonicity [47], causality, additivity [48], or domain-specific physical constraints. For structured data, sparsity is often a valuable measure of interpretability, given that humans can cognitively process only three to five entities simultaneously [49]. Sparse models facilitate an understanding of how variables interact collectively rather than in isolation.

Given these considerations, this study aims to develop an ante hoc method that addresses key challenges in QXAI. Specifically, this research will focus on:

- Formulating an effective strategy for disentangling and representing concepts within a QNN model.
- Ensuring that the concepts are sparse and independent of each other.
- Designing a QNN that seeks to balance high predictive accuracy with interpretability.

To tackle these challenges, this study proposes the development of the concept-driven quantum neural network (CD-QNN) model. The CD-QNN model aims to maintain high predictive accuracy while providing clear and meaningful explanations for its decisions. By integrating the strengths of QNNs with the interpretability of self-explanatory models, CD-QNN maps input data into a human-understandable concept space and bases its decisions on these concepts. This approach not only bridges the gap between the computational power of QNNs and the need for model interpretability but also enhances the trustworthiness and reliability of quantum artificial intelligence systems.

To ensure that the explanations provided by the CD-QNN model are direct and easily understandable, measures have been implemented to align these explanations closely with the model’s behavior, accurately reflecting the true importance of features in the decision-making process. It is crucial to maintain the consistency and reliability of the explanations, ensuring that the model generates similar explanations for similar input samples. This consistency allows users to quickly grasp the basis of the model’s decisions, thereby enhancing the model’s transparency and credibility.

The contents of this paper are organized as follows. Section 2 reviews the pertinent literature and identifies gaps in the current research. Section 3 examines the design and implementation of the concept generator, demonstrating how abstract, human-interpretable concepts are derived from raw input data using a QVAE. Section 4 outlines a detailed algorithmic framework for CD-QNN, covering the *concept generator*, *feature extractor*, and *feature integrator*. Section 5 focuses on optimizing the training strategy to enhance and balance classification performance and interpretability. Section 6 presents the experimental results validating the effectiveness of CD-QNN, emphasizing its predictive accuracy and the clarity of its model explanations. Finally, Section 7 summarizes this study’s contributions and proposes directions for future research.

## 2. Related Works

The field of Quantum Neural Networks (QNNs) has seen significant advancements in recent years, driven by the potential of leveraging quantum computing to solve complex problems more efficiently than classical methods [12]. Numerous studies have explored the computational advantages and applications of QNNs across various domains [50,51,52].

One of the foundational developments in this area is the quantum variational algorithm, which employs quantum circuits with parameterized gates that can be optimized similarly to neural networks in classical machine learning [53]. Farhi et al. [14] introduced the Quantum Approximate Optimization Algorithm (QAOA), demonstrating the potential of quantum circuits to solve combinatorial optimization problems. Similarly, the Variational Quantum Eigensolver (VQE) has been widely used in quantum chemistry to find the ground state energies of molecules [15]. These algorithms laid the groundwork for integrating quantum computing with neural network architectures [54].

Building on these concepts, several researchers have proposed quantum versions of classical neural network models. For instance, Schuld et al. [55] developed a quantum perceptron model, highlighting the feasibility of implementing neural network operations on quantum computers. Subsequent works extended these ideas, introducing quantum convolutional neural networks [17,56]. These studies have demonstrated that QNNs can outperform their classical counterparts in specific tasks, especially as quantum hardware continues to advance [57].

Despite these promising developments, a significant challenge in the field of QNNs is the interpretability of the models. Most existing QNNs function as black boxes, making it difficult for users to understand the decision-making processes. This lack of transparency hinders the adoption of QNNs in critical applications where interpretability is essential, such as healthcare and finance [27].

Traditional methods in classical machine learning to enhance the interpretability include techniques like saliency maps [32,58,59], LIME (Local Interpretable Model-agnostic Explanations) [31], and SHAP (SHapley Additive exPlanations) [60]. These methods aim to provide insights into how individual features contribute to the model’s predictions. Directly applying these techniques to QNNs is not straightforward due to the intrinsic differences between classical and quantum data representations [13].

In the context of quantum computing, a few pioneering studies have attempted to introduce interpretability. Burge et al. [42] introduced a quantum algorithm to estimate Shapley values, facilitating fair payoff distribution in cooperative game scenarios through innovative beta function approximations. Heese et al. [43] extended classical Shapley values to quantum circuits, developing PolynomialSHAP for feature importance in Quantum Machine Learning (QML) models, demonstrating robustness across simulated and real quantum environments. Steinmüller et al. [46] explored ways to accelerate the computation of Shapley values using the internal mechanics of QNNs. Pira et al. [45] proposed Quantum LIME, an adaptation of classical techniques to the quantum domain. Mercaldo et al. [44] investigated QML applications in mobile malware detection, highlighting the critical role of explainability in security contexts. Collectively, these studies enhance the transparency and trustworthiness of QML, fostering its adoption in practical applications.

## 3. Concept Generator

In traditional interpretable model frameworks, each input variable is typically treated as a fundamental unit in the explanation process. While this approach is technically rigorous, it does not fully align with how humans process information. For example, when interpreting an image, humans do not rely on individual pixels to explain the content; instead, they use more abstract, high-level concepts such as object contours or brushstroke thickness. Similarly, in the quantum realm, a single qubit state can be compared to a pixel in computer vision, while the relationships and entanglements between multiple qubits represent higher-order, human-understandable concepts.

To incorporate these concepts into the model instead of relying on raw inputs, we mathematically define a mapping function $c\left(\cdot \right):X\to C\subset R^{k}$, where X represents the original input space, and C represents the space composed of human-interpretable concepts. To ensure sparsity, these concepts should be simple and easy for humans to understand, with the number of concepts *k* controlled within a small range.

The construction of the mapping function c(·), or the abstraction of key concepts from raw inputs, must not only provide sufficient information for subsequent model judgments but also ensure that these concepts are intuitive and verifiable from a human perspective. One method to achieve this is through predefined feature extractors, typically built based on domain experts’ knowledge [61,62,63]. This approach often requires customized feature extractors for different domains, which can be costly and lack general applicability. An alternative approach is to learn latent space representations to extract concepts and impose specific constraints on these representations to ensure that the extracted concepts have practical significance [64,65].

When constructing a conceptualized model, it is essential to focus on the following core attributes:

1. The critical information in the input data should be maximally captured by the abstracted concepts. This can be achieved by defining c(·) as the encoder part of a quantum variational autoencoder (QVAE) [66]. In this architecture, the input x is transformed into a lower-dimensional representation by the encoder c(·) and then reconstructed by the decoder d(·), i.e., $\hat{x}=d\left(c\left(x\right)\right)$. This low-dimensional latent space representation encapsulates the most critical information contained in the input x.
2. To ensure both sparsity and disentanglement, the input data should be representable by a finite and non-overlapping set of concepts. This can be accomplished through the sparsity control of the QVAE. A sparse autoencoder activates only a small part of the latent space dimensions for a given input. Increasing sparsity encourages the model to represent the input with fewer concepts while leveraging the advantages of β-VAE can make the latent space representations more inclined toward disentanglement and independence.
3. The extracted concepts need to be intuitively interpretable by humans. This can be achieved through *prototype interpretation* of concepts. By varying $c{\left(x\right)}_{i}$ for the same data and inputting the altered latent space representation into the decoder to obtain reconstructed data, the impact of concept representation changes on the reconstructed data can be observed. This method of interpreting concepts is referred to as prototype interpretation.

### 3.1. Designing QVAE as a Concept Generator

Existing research on QVAE [66] primarily employs energy models [67], such as quantum Boltzmann machines [68,69], as decoders. This reliance on specific quantum models limits QVAE’s flexibility in training and scalability, particularly in leveraging contemporary deep neural network technologies. Therefore, this study aims to redesign the QVAE architecture to enhance its efficiency and scalability, making it more suitable as a concept generator in CD-QNNs.

**Figure 1:**
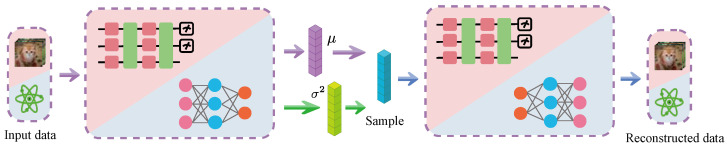
Model architecture diagram of QVAE used to train the concept generator in CD-QNN. This figure illustrates the architecture of the Quantum Variational Autoencoder (QVAE) used to generate human-interpretable concepts in CD-QNN. The purple and green vectors represent the mean and variance outputs from the decoder, respectively. These are used to sample a latent vector, depicted in blue, which serves as a one-dimensional vector. Each element of this vector corresponds to a distinct concept, which is used in the subsequent model stages.

The model architecture of QVAE used to train the concept generator is shown in Figure 1. Inspired by the flexibility of quantum adversarial neural networks in independently selecting models for generators and discriminators [18,70,71], the encoder and decoder of the newly designed QVAE can independently choose between quantum or classical models.

When a quantum neural network (QNN) is selected as the encoder and a deep neural network (DNN) as the decoder, the QVAE functions as a quantum-classical hybrid model. To clearly distinguish between the VAE’s encoder and the process of loading classical data into quantum states, the latter is referred to as the *dataloader*.

The encoder utilizes a hardware-efficient ansatz, which enhances the expressivity of the quantum circuit through a series of parameterized unitary transformations [72]. This approach employs a layered structure, where each layer consists of parameterized rotation gates (based on Pauli X, Y, and Z matrices) and entanglement gates (such as CNOT gates), enabling efficient quantum computation while ensuring compatibility with current quantum hardware limitations [57]. The adjustable parameters within these rotation gates allow the quantum circuit to adapt and optimize for specific quantum tasks during the learning process. Quantum state measurements and quantum information processing are primarily conducted using orthogonal measurements on a computational basis. These measurements are crucial for the effective conversion and processing of quantum-classical information required for downstream tasks [73].

Mathematically, the quantum encoder can be represented as follows. First, a dataloader $U_{x}\left(x\right)$ encodes classical data x into a quantum state |ψ(x)〉:

(1) $$|\psi (x)\rangle =U_{x}\left(x\right){|0\rangle }^{⊗n}.$$

This encoding can typically be achieved using rotation gate dataloaders, amplitude dataloaders, or data re-uploading dataloaders [74,75,76]. For instance, using a rotation gate dataloader, the classical data x can be mapped to a quantum state as follows:

(2) $$U_{x}\left(x\right){|0\rangle }^{⊗n}=\overset{n}{\underset{i=1}{⨂}}R_{y}\left(x_{i}\right)|0\rangle ,$$

where $R_{y}\left(x_{i}\right)=e^{-ix_{i}Y/2}$ represents the rotation around the Y-axis by angle $x_{i}$, and ${|0\rangle }^{⊗n}$ is the initial state of *n* qubits, all set to |0〉.

The quantum circuit is then parameterized as

(3) |ϕ(x;θ)〉=U(θ)|ψ(x)〉,

where U(θ) represents the hardware-efficient ansatz with parameters θ. Specifically, U(θ) can be decomposed into a sequence of parameterized single-qubit rotation gates and entangling gates, as follows:

(4) $$\begin{array}{cc} U(\theta ) & =\prod_{l=1}^{L}U_{l}\left(\theta_{l}\right) \end{array}$$

(5) $$\begin{array}{cc} U_{l}\left(\theta_{l}\right) & =\prod_{q=1}^{n}U_{l,q}\left(\theta_{l,q}\right)U_{ENT} \end{array}$$

(6) $$\begin{array}{cc} U_{l,q}\left(\theta_{l,q}\right) & =R_{z}\left(\theta_{l,q}^{\left(1\right)}\right)R_{x}\left(\theta_{l,q}^{\left(2\right)}\right)R_{z}\left(\theta_{l,q}^{\left(3\right)}\right) \end{array}$$

(7) $$\begin{array}{cc} U_{ENT} & =\prod_{q=1}^{n}\mathrm{CNOT}\left(q,\left(q+1\right)\;\mathrm{mod}\;n\right), \end{array}$$

where $R_{x}\left(\theta \right)$ and $R_{z}\left(\theta \right)$ are rotation gates around the X and Z axes, respectively, applied to qubit *q* in layer *l*, and CNOT(q,(q+1)modn) represents a controlled-NOT gate acting on qubits *q* and (q+1)modn.

Finally, the measurement operation collapses the quantum state to a classical vector $z=[z_{1},z_{2},\ldots ,z_{n}]$, as follows:

(8) $$z_{i}=\langle \phi \left(x;\theta \right)|{\hat{Z}}_{i}|\phi \left(x;\theta \right)\rangle ,$$

where ${\hat{Z}}_{i}$ is the Pauli-Z operator acting on the *i*-th qubit, and $z_{i}$ represents the expectation value of the measurement outcome for qubit *i*.

Unlike traditional autoencoders that map inputs directly to fixed points in the latent space, variational autoencoders (VAEs) map inputs to probability distributions in the latent space. Following the quantum model output, two classical fully connected layers estimate the mean and variance of the probability distribution, as follows:

(9) $$\mu ={\mathrm{FC}}_{1}\left(z\right),\;\sigma ={\mathrm{FC}}_{2}\left(z\right),$$

where ${\mathrm{FC}}_{1}$ and ${\mathrm{FC}}_{2}$ are fully connected layers. The reparameterization trick is then applied to ensure differentiability:

(10) $$\tilde{z}=\mu +\sigma \cdot \epsilon ,\;\epsilon ∼N\left(0,I\right).$$

Subsequently, a DNN serves as the decoder. The decoder network, represented as d(·), maps the latent variable $\tilde{z}$ back to the original input space, as follows:

(11) $$\hat{x}=d\left(\tilde{z}\right).$$

The overall loss function for training the QVAE combines reconstruction loss and a regularization term to enforce a prior distribution on the latent space, as follows:

(12) $$L=E_{q_{\phi }\left(z|x\right)}\left[\log p_{\theta }\left(x|z\right)\right]-D_{KL}\left(q_{\phi }\left(z|x\right)∥p\left(z\right)\right),$$

where $D_{KL}$ represents the Kullback–Leibler divergence.

#### Conversion Between Quantum and Classical Data

It should be noted that the conversion between quantum and classical data inevitably results in resource consumption and potential information loss. For input data originally in the form of quantum states, it can be directly processed by the quantum encoder. Classical data can be converted to quantum states using dataloaders. In practical applications, selecting the appropriate QVAE configuration to balance computational efficiency and accuracy is crucial. By simply modifying the encoder, it can be adapted to function as a DNN. Similarly, the decoder can be transformed into a QNN. This flexibility allows the concept generator to handle both quantum and classical data, achieving seamless integration and enhancing the model’s versatility.

### 3.2. Sparse and Disentangled Representations in QVAE

Typically, the divergence of the encoder’s output from a standard Gaussian distribution is measured using the KL divergence, which is then minimized. To encourage the encoder to generate sparse latent space variables, an L1 regularization term can be added to the autoencoder’s loss function. This approach reduces redundancy between features and aims to decouple concepts as much as possible, as follows:

(13) $$L_{\mathrm{total}}=L+\lambda \sum_{i=1}^{n}\left|z_{i}\right|.$$

Merely enforcing the activation of a subset of the input data’s dimensions does not guarantee the independence of concepts. Each concept may still represent a complex and intertwined set of features, complicating the interpretation of each individual concept learned by the model. To address this issue more effectively, the loss function of β-VAE [77] is adopted as a framework for learning disentangled concepts. In the β-VAE model, an adjustable hyperparameter β introduces stronger constraints on the latent space by increasing the penalty on KL divergence. As the value of β increases, the latent space is encouraged to approach a unit Gaussian distribution. Given the independence of dimensions in a unit Gaussian distribution, this mechanism also promotes the disentanglement of latent factors, as follows:

(14) $$L=E_{q_{\phi }\left(z|x\right)}\left[\log p_{\theta }\left(x|z\right)\right]-\beta D_{KL}\left(q_{\phi }\left(z|x\right)∥p\left(z\right)\right).$$

By carefully adjusting β, CD-QNN ensures that the latent space is sufficiently disentangled to produce interpretable concepts while preserving important information and maintaining high reconstruction fidelity. As demonstrated in our experiments, increasing β improves disentanglement without significantly degrading reconstruction quality or predictive performance. In addition, sparsity is selectively applied to activate only the most relevant concepts, reducing redundancy and focusing the model on essential features. This approach aligns with human cognitive processing, making the model more interpretable without sacrificing expressiveness. Through our ablation studies, we show that even with sparsity constraints, CD-QNN maintains high classification accuracy, confirming that critical quantum information is retained.

### 3.3. Prototype Interpretation

Utilizing the properties of the QVAE, it is possible to generate continuously varying data prototypes. Unlike the original inputs of the training dataset, these prototypes can freely navigate within the dimensional space of the concepts. By analyzing the reconstructed data from these continuous variations, a more comprehensive understanding of the concept’s significance can be attained, thereby enabling the exploration and visualization of the specific content of the concepts. This method not only deepens our grasp of the concepts themselves but also enhances our comprehension of how the model discerns and differentiates these concepts. Consequently, this approach offers new insights into the intrinsic structure of data and the decision-making logic of the model.

### 3.4. Mitigating KL Divergence Vanishing

During the training of QVAE, an issue known as the KL divergence vanishing problem was identified. This phenomenon occurs when the Kullback–Leibler (KL) divergence term, which serves as the regularization component, approaches zero throughout the training process. The KL divergence encourages the latent representation to conform to a prior distribution, typically a standard normal distribution. When the KL divergence diminishes, it suggests that the model may neglect the structure of the latent space, focusing solely on minimizing reconstruction error. This oversight results in a lack of meaningful structure within the latent space, thereby adversely impacting the model’s performance and generalization capabilities.

To mitigate this issue, additional configurations were incorporated into the QVAE model, as suggested by [78]. Specifically, Batch Normalization layers were integrated to standardize the mean and variance of the latent space, thereby stabilizing and accelerating the training process. Subsequently, the mean and variance were adjusted using the following scaling layer:

(15) $$\begin{array}{cc} \mu & =\sqrt{\tau +(1-\tau )\cdot \mathrm{sigmoid}(\theta )}\mu , \end{array}$$

(16) $$\begin{array}{cc} \sigma & =\sqrt{\left(1-\tau )\cdot \mathrm{sigmoid}(-\theta \right)}\sigma \end{array}$$

where τ is a hyperparameter ranging between 0 and 1, and θ is a trainable parameter. This approach enhances the model’s responsiveness to the KL divergence by modifying the latent space scale, thereby addressing the KL divergence vanishing problem. Subsequent experiments demonstrated that incorporating this scaling layer effectively improves the model’s training speed, albeit at the cost of a slight increase in QVAE’s reconstruction loss.

## 4. CD-QNN Architectural Design

**Figure 2:**
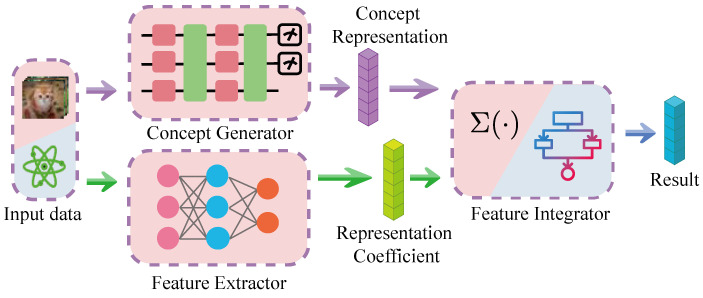
This figure illustrates the architecture of CD-QNN. The purple vectors represent the concept representations, while the green vectors indicate the representation coefficients. Together, these elements are used by the feature integrator to make the final prediction.

The CD-QNN model is composed of three fundamental components: a concept generator *c*, a feature extractor θ, and a feature integrator *g*. The comprehensive architecture is depicted in Figure 2. In this structure, the concept generator compresses the raw data into a sparse conceptual representation, which can be realized through the encoder of a QVAE. This encoder converts raw data into a conceptual representation comprehensible to humans. The feature extractor, typically realized by a DNN, is tasked with extracting pertinent information from the raw data to ensure adequate expressivity. The feature integrator amalgamates various concepts into the final output. To preserve the model’s interpretability, the feature integrator should be as simplistic as possible. This simplicity aids in understanding each concept’s contribution to the final output.

Achieving a balance between the algorithm’s interpretability and expressivity is crucial. The deep learning model’s expressivity refers to its ability to represent and approximate complex functions. This capability is influenced by factors such as the number of parameters, network depth, width, and the choice of activation functions. Quantitative analysis of expressivity includes evaluating parameter count and capacity, the Vapnik–Chervonenkis (VC) dimension [79], and empirical studies on generalization error versus training error [80]. Theoretical approaches, such as the Universal Approximation Theorem [81], and practical methods, including pruning and analyzing information bottlenecks [82], provide insights into a model’s expressivity. Recent studies also highlight the potential of quantum-inspired enhancements for generative models, where quantum correlations provide a powerful resource for improved expressivity [83]. Moreover, the expressivity of variational quantum algorithms has been analyzed using advanced tools in statistical learning theory, revealing that the number of quantum gates and measurement observables upper bound expressivity, with implications for the trainability and generalization of QNNs [84].

This equilibrium between interpretability and expressivity is well-reflected in the selection of three components. The choice of an appropriate model is contingent on the relative importance of interpretability and expressivity. The flexibility of the CD-QNN design lies in its modularity. Each component—the concept generator, feature extractor, and feature integrator—can be independently selected and configured based on specific application requirements. This modular approach allows for tailored adjustments to optimize the balance between representational capacity and interpretability. For instance, in applications where interpretability is paramount, simpler models for the feature integrator and concept generator can be chosen. Conversely, for tasks demanding higher expressivity, more complex models can be employed, ensuring the model remains robust and versatile across diverse scenarios. This design philosophy underscores the adaptability of CD-QNN, making it a powerful framework for integrating quantum and classical computing paradigms in machine learning.

The ensuing discussion will delve into the design methodologies of the model architecture, commencing with the most elementary linear models and gradually advancing towards more intricate constructs. Linear models possess the inherent advantage of interpretability. By methodically abstracting and complicating them, the model’s expressivity can be enhanced. The challenge in this process lies in retaining the model’s essential interpretability while augmenting its expressivity.

### 4.1. Linear Models

Consider a set of input features $x_{1},\ldots ,x_{n}$ and their corresponding parameters $\theta_{1},\ldots ,\theta_{n}$. The prediction of a linear model can be concisely represented as

(17) $$f\left(x\right)=\theta^{T}x=\sum_{i=1}^{n}\theta_{i}x_{i}.$$

For simplicity, the bias term is temporarily disregarded in this model. This model is considered highly interpretable due to the following conditions:

1. There exists a direct and explicit relationship between the input features and the model’s decision-making process.
2. Each parameter $\theta_{i}$ quantitatively reflects the influence of its corresponding feature $x_{i}$ on the prediction outcome, with the sign of $\theta_{i}$ indicating whether this influence is positive or negative.
3. The overall prediction output of the model is a linear aggregation of the influences of each feature, ensuring the clarity of each feature’s impact on the prediction. There is no interaction between features, thus avoiding any ambiguity in interpretation.

Building upon this foundation, we will incrementally extend the linear model to explore how to preserve the integrity of interpretability mechanisms as the model’s complexity escalates, aiming to enhance the model’s expressivity while maintaining the transparency of the decision-making process.

### 4.2. Feature Extractor

To augment the expressivity of the linear model while preserving its overall structure, the model’s coefficients can be dynamically adjusted based on the input x. This adjustment can be mathematically represented as $f\left(x\right)=\theta {\left(x\right)}^{T}x$, where θ is derived from a model space Θ, which encompasses a range of models, from conventional regression models to DNNs. In the absence of additional constraints, this dynamic coefficient model can achieve functionalities akin to those of DNNs. For multi-class classification tasks, θ(x) is a $k\times n_{cl}$ matrix, where *k* denotes the input dimension and $n_{cl}$ denotes the number of classes.

To ensure interpretative stability, it is imperative to guarantee that the variations in coefficients θ(x) and $\theta (x^{\prime })$ for similar inputs x and $x^{\prime }$ are minimal. By incorporating regularization terms, such as enforcing $\nabla_{x}f\left(x_{0}\right)\approx \theta \left(x_{0}\right)$ in the vicinity of $x_{0}$, the model can locally approximate a linear model with stable coefficient vectors $\theta (x_{0})$. This design ensures that the modifications in coefficients $\theta_{i}\left(x\right)$ remain comprehensible, allowing them to dynamically adjust according to variations in input x, but in a relatively gradual manner.

This regularization terms serve to control the fluctuations in coefficients steadily, guiding the model to effectively extract pertinent information from complex data, thus preventing overfitting while maintaining interpretability. The meticulous selection and design of regularization techniques are crucial in balancing model interpretability and performance, influencing the model’s behavior at individual points $x_{i}$ as well as its generalization capability across the entire input space.

### 4.3. Concept Generator

To transition from raw data to human-understandable concepts, the encoder of a QVAE is employed to derive these *concept representations*, denoted as c(x). By utilizing these methodologies, an interpretable model has been developed, as follows:

(18) $$f\left(x\right)=\theta {\left(x\right)}^{T}x=\sum_{i=1}^{k}\theta {\left(x\right)}_{i}c{\left(x\right)}_{i},$$

where each $c{\left(x\right)}_{i}$ is a numerical scalar representing the degree of presence of a specific idea in the input x. These corresponding coefficients are referred to as *representation coefficients*. The *concept contribution* is obtained by multiplying the concept representation with the representation coefficient, determining the contribution of the concept to the class prediction. If a sample has a negative representation coefficient for a certain concept, it indicates a weak expression of that concept in the sample; if the corresponding concept representation is also negative, it implies that a weak representation of that concept is needed for the sample to belong to the current class. Then the product of these two values results in a positive concept contribution, indicating that the concept positively contributes to the class determination.

For example, considering the idea of brushstroke thickness, the value of $c{\left(x\right)}_{i}$ can range from −1 to 1, where −1 indicates extremely thin brushstrokes and 1 indicates extremely thick brushstrokes. The identification of the strength of these ideas, obtained through the encoder, and their corresponding coefficients in the samples is achieved through a learning process. This process ensures that each representation accurately reflects the nuances of the input data.

### 4.4. Feature Integrator

A feature integrator *g* is introduced to thoroughly process the transformed features and generated concepts. Specifically, the final form of the model can be expressed as

(19) $$f\left(x\right)=g(\theta {\left(x\right)}_{1}c{\left(x\right)}_{1},\ldots ,\theta {\left(x\right)}_{k}c{\left(x\right)}_{k}),$$

where *g* is a function that retains the model’s interpretability attributes.

Linear models are an intuitive and widely utilized choice for feature integrators due to their interpretability and simplicity. By linearly combining various features, the contribution of each feature to the final result can be directly observed. Generalized linear models extend linear models by allowing the response variable’s distribution to belong to the exponential family and linking the linear predictor to the expected response through a link function [34]. These models perform well when dealing with data exhibiting specific distribution characteristics while maintaining a degree of interpretability. Decision trees represent another viable option. They construct a tree-like structure to partition the feature space into different regions and make decisions within each region [35,36]. The advantage of decision trees lies in their clear structure, which simplifies the decision process. The splitting conditions at each node and the output results at leaf nodes possess explicit physical meanings, aiding in the explanation and analysis of the model.

In practical applications, the appropriate feature integrator can be selected based on the data characteristics and model requirements. By making an informed choice, the prediction accuracy and generalization capability of the model can be enhanced while ensuring its interpretability.

## 5. Training Strategy

Building on the previous analysis and design, a two-stage training strategy is proposed to balance the model’s pursuit of interpretability and classification performance. Since both the concept generator and feature extractor are functions of the input data, training these two components simultaneously would conflate the degree of expression of concepts (latent space representation of the concept generator) with the relevance coefficients of concepts in the model’s judgment (output of the feature extractor). Therefore, a *two-stage* training process is essential. Empirical evidence indicates that concurrent training of the concept generator and feature extractor can lead to model collapse.

In the first stage, the focus is on training the concept generator to acquire the latent space representations of the input data. The second stage involves training the feature extractor and feature integrator while keeping the parameters of the concept generator fixed. If a linear model is selected as the feature integrator, optimization is unnecessary.

### 5.1. Concept Generator Loss

The objective of optimizing the concept generator is not only to accurately reconstruct the initial input with the generated concepts but also to ensure the orthogonality of the concepts so that the latent variables in each dimension are as independent as possible. To meet this requirement, a concept loss $C_{c}\left(x\right)$ is introduced. Within the β-VAE framework, this loss function is mathematically expressed as

(20) $$C_{c}\left(\phi ,\psi ;x,\beta \right)=E_{q_{\phi }\left(z|x\right)}\left[\log p_{\psi }\left(x|z\right)\right]-\beta D_{KL}\left(q_{\phi }\left(z|x\right)∥p\left(z\right)\right),$$

where ϕ and ψ denote the parameter sets of the encoder and decoder, respectively. The first term represents the reconstruction error, measuring the model’s accuracy in reconstructing the input data through latent variables, while the second term is the KL divergence regularization term, measuring the deviation of the latent variable distribution from its prior distribution, with β being a hyperparameter controlling the balance between the two terms.

KL (Kullback–Leibler) divergence measures the difference between two probability distributions. In VAE, the goal is to learn a latent space distribution that approximates a standard normal distribution as closely as possible. The formula for KL divergence in this context is

(21) $$D_{KL}\left(q_{\phi }\left(z|x\right)∥p\left(z\right)\right)=\frac{1}{2}\sum_{j=1}^{J}\left(\mu_{j}^{2}+\sigma_{j}^{2}-\log\left(\sigma_{j}^{2}\right)-1\right),$$

where $q_{\phi }\left(z|x\right)$ is the distribution of latent variables z output by the encoder, p(z) is the desired standard normal distribution, and $\mu_{j}$ and $\sigma_{j}$ are the mean and variance of the latent variables. This formula is derived by calculating the expected values and logarithmic differences in the two distributions. In practice, the KL divergence of each sample is summed and averaged to obtain the KL divergence for the entire batch.

### 5.2. Classification Loss

To ensure the model’s accuracy in handling real datasets, a classification loss $C_{\theta }\left(x,y\right)$ is introduced, reflecting the consistency between the model’s predictions and the true labels. In classification problems, the cross-entropy loss function is commonly used to evaluate model performance. Assuming the model’s output f(x) is the predicted probability distribution for each class and *y* is the true label distribution, usually in one-hot encoding form, the mathematical expression of the cross-entropy loss function is

(22) $$C_{\theta }\left(x,y\right)=-\sum_{i}y_{i}\log\left(f_{i}\left(x\right)\right),$$

where $y_{i}$ indicates the presence of the true label in class *i* and $f_{i}\left(x\right)$ represents the model’s predicted probability for class *i*. For binary classification problems, this loss function can be further simplified to

(23) $$C_{\theta }\left(x,y\right)=-\left[y\log\left(p\right)+\left(1-y\right)\log\left(1-p\right)\right]$$

where *p* is the model’s predicted probability of the positive class and *y* is the binary true label (0 or 1).

### 5.3. Stability of Interpretations

To ensure the stability of the generated interpretations, constraints on the model’s continuity and local stability are imposed. Assuming $\theta_{i}$ is a constant parameter and treating the function *f* as a function of c(x), i.e., f(x)=g(c(x)), and let z=c(x). Applying the chain rule, we obtain $\nabla_{x}f=\nabla_{z}f\cdot J_{c}\left(x\right)$, where $J_{c}\left(x\right)$ represents the Jacobian matrix of *c* with respect to x. At a data point $x_{0}$, we want $\theta (x_{0})$ to approximate the derivative of *f* with respect to the concept vector c(x) at least locally, i.e.:

(24) $$∥\nabla_{x}f\left(x\right)-\theta {\left(x\right)}^{T}J_{c}\left(x\right)∥\approx 0.$$

Based on this, the difference can be included as a regularization term R(x) in the following loss function:

(25) $$R\left(x\right):=∥\nabla_{x}f\left(x\right)-\theta {\left(x\right)}^{T}J_{c}\left(x\right)∥.$$

It should be noted that involving the calculation of second-order derivatives will significantly increase the complexity of model training. Empirical studies have shown that in certain scenarios, the interpretations themselves exhibit good stability, so the decision to introduce this regularization term should be balanced against training resources and the stability performance in the specific scenario.

## 6. Experiments

In this section, we present a comprehensive evaluation of the Concept-Driven Quantum Neural Network (CD-QNN) model through a series of meticulously designed experiments. The DIGIT dataset is utilized to train, validate, and test the model, employing various configurations to assess performance. We conduct ablation studies to investigate the impact of different parameters and components on the model’s classification accuracy, reconstruction error, and interpretability. By examining multiple experimental setups, we aim to highlight the strengths and limitations of the CD-QNN, providing insights into the effectiveness of the two-stage optimization strategy and the role of quantum-classical hybrid models in enhancing machine learning frameworks. This section is structured to present the preprocessing steps, training methodologies, detailed results from ablation studies, and in-depth analyses of classification accuracy, reconstruction error, and prototype interpretation.

### 6.1. Experiment Setup

The DIGIT dataset was preprocessed and partitioned into training, validation, and test sets in proportions of 72%, 8%, and 20%, respectively. During the training phase, the Adam optimizer was employed with an initial learning rate of 0.001, β parameters of (0.9,0.999), ϵ set at $1\times 10^{-8}$, without weight decay, and AMSGrad was not utilized [85,86]. The training process continued for multiple epochs until the model’s performance met the desired criteria or the stopping conditions were met.

As detailed in Section 3.1, the QVAE model was trained using a quantum encoder and a classical decoder, with the quantum encoder subsequently employed as the concept generator. The specific configuration of the QVAE is outlined in Table 1. The binary cross-entropy loss function was applied, thus normalization operations were omitted during data preprocessing. The loss function comprises both reconstruction loss and KL divergence, with their balance managed by adjusting the hyperparameter β. This adjustment facilitates a trade-off between reconstruction accuracy and the smoothness of the latent space. It is critical that the reduction method for both components of the loss function remains consistent, either using the sum or mean, to avoid instability during training.

In the classical decoder, LeakyReLU was employed for activation functions to mitigate the vanishing gradient problem associated with the negative half-axis of ReLU [87]. The LeakyReLU function is defined as

(26) $$f\left(x\right)=\left\{\begin{array}{cc} x & \mathrm{if}\;x>0,\\ \alpha x & \mathrm{if}\;x\leq 0, \end{array}\right.$$

where α is a small positive value, set at 0.2 for this experiment. LeakyReLU introduces a slight slope in the negative region, effectively addressing the issue of zero gradients in ReLU activation functions. The classical decoder configuration ensures the output remains within the [0,1] range by incorporating a Sigmoid activation function in its final layer.

The feature extractor comprises two convolutional neural network layers, each utilizing [10,20] 3×3 convolutional kernels, followed by two fully connected layers containing [160,80] neurons, respectively. To enhance the generalization capability of the feature extractor, dropout layers with a parameter of 0.2 were included during training. The feature integrator employs a simple summation function without any trainable parameters.

The aforementioned configuration is not optimized. Therefore, a series of ablation studies were designed to further explore the impact of various modules and parameters on the CD-QNN model’s performance. The following aspects were considered:

- Adjusting the β value allows observation of the balance between reconstruction accuracy and latent space smoothness. Increasing β enhances the constraint on the latent space, which may reduce reconstruction accuracy slightly but results in a smoother latent space.
- Modifying the number of measurements or layers in the QNN affects model performance. Reducing measurements can complicate latent space representation extraction, whereas increasing network layers enhances learning capacity but also adds training complexity.
- Introducing a scaling layer adjusts feature scales to improve model generalization and stability. Omitting the scaling layer may cause feature values to be excessively large or small, impacting model convergence speed and performance.
- Employing different dataloaders, such as rotation gate and amplitude dataloaders, alters the model’s expressivity and learning dynamics, thereby influencing final performance.
- The choice between the two-stage optimization strategy proposed herein and the simultaneous optimization of the concept generator and feature extractor significantly impacts the training process.

Based on these considerations, nine sets of experiments were conducted, each altering only one or two configurations, as summarized in Table 2.

### 6.2. Classification Accuracy

**Figure 3:**
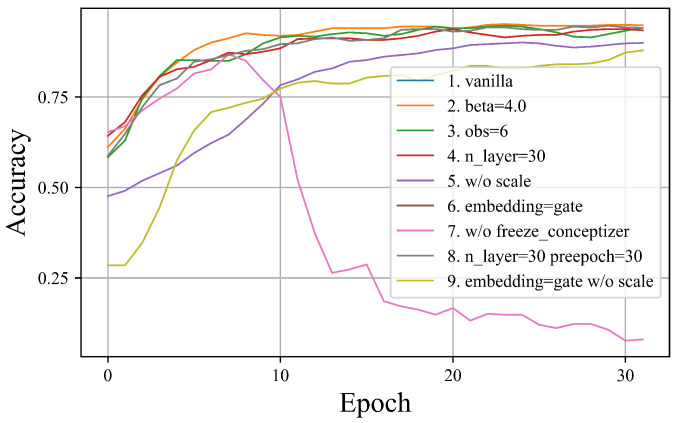
Comparison of classification accuracy for CD-QNN under different configurations.

The classification accuracy of CD-QNN models was assessed, and the outcomes are depicted in Figure 3. This figure illustrates the accuracy trajectory throughout the training process, with the horizontal axis representing the number of training epochs and the vertical axis indicating accuracy. Upon comparing the results across various configurations, several conclusions can be inferred:

- Group 7 reveals that employing a mixed training strategy, where the concept generator is trained concurrently with the feature extractor, enhances the model’s accuracy in the initial phases. Nevertheless, as training advances, the model eventually deteriorates. Experiments involving the concept generator with rotation gate dataloaders produced similar outcomes, demonstrating that irrespective of the model architecture, the mixed training strategy ultimately results in model failure. This underscores the significance of the two-stage training strategy, wherein the feature extractor is trained first, followed by the concept generator, to maintain model stability and efficacy.
- Omitting the scaling layer in the latent space influences the model’s training speed. Specifically, the rate of accuracy improvement during training is diminished, potentially because the scaling layer assists in adjusting the feature space distribution, thereby expediting model convergence. Although the convergence rate is reduced, the CD-QNN without the scaling layer can still achieve over 95% accuracy with an increased number of training iterations.
- Aside from the aforementioned factors, modifications in other configurations exert relatively minor impacts on model accuracy, generally achieving approximately 95% accuracy. It is noteworthy that subsequent interpretability analyses can disclose significant differences in interpretability even between two models with identical accuracy. This finding further highlights the limitation of relying solely on a single metric to evaluate model performance and the necessity to consider various aspects, particularly the model’s internal decision-making mechanisms.

### 6.3. Reconstruction Error

**Figure 4:**
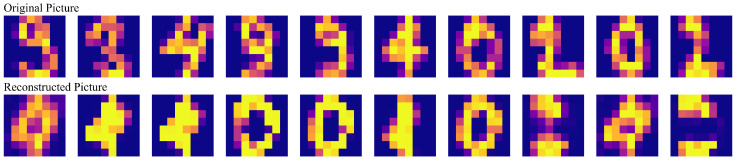
Reconstruction of images from the validation set in Group 2 (beta = 4).

**Figure 5:**
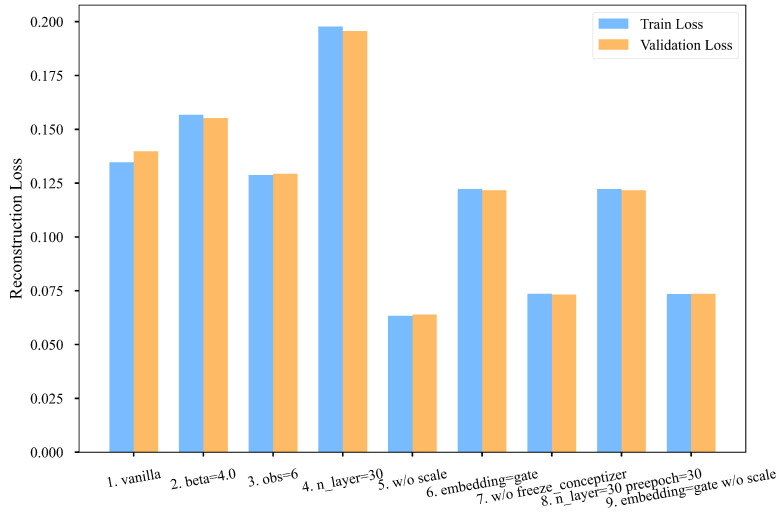
Comparison of reconstruction loss after the final training iteration.

To further assess model performance, the reconstructions of 10 samples from Group 2 are presented in Figure 4. For a more detailed analysis, the reconstruction loss of various configurations was compared after the final training iteration. Reconstruction loss was quantified using mean squared error (MSE), as illustrated in Figure 5. Comparing these nine groups, the following conclusions were derived:

- None of the models exhibited overfitting, indicating they maintained robust generalization throughout the training process.
- Comparing Groups 1, 4, and 8, it was observed that increasing the number of layers in the quantum network enhances the model’s expressivity, albeit at the cost of increased training resources and time.
- The comparison between Groups 1 and 2 revealed that increasing the weight coefficient β of the reconstruction error may lead to higher reconstruction loss, necessitating a balance in practical applications.
- The comparison between Groups 1 and 3 demonstrated that a 10-layer QNN, with only 180 trainable parameters, can effectively compress a 64-dimensional vector to 6 dimensions (through 6 measurements) while achieving good reconstruction accuracy.
- The comparisons among Groups 1, 5, 6, and 9 indicated that regardless of using rotation gate dataloaders or amplitude dataloaders, removing the scaling layer in the latent space can reduce reconstruction loss.
- The comparison between Groups 1 and 6 showed that both rotation gate dataloaders and amplitude dataloaders enable the QNN to effectively extract information.
- The comparison between Groups 1 and 7 indicated that training the concept generator concurrently with the feature extractor reduces reconstruction loss due to the increased number of iterations. However, the model eventually collapses, resulting in blurred reconstructed images.

### 6.4. Prototype Interpretation

**Figure 6:**
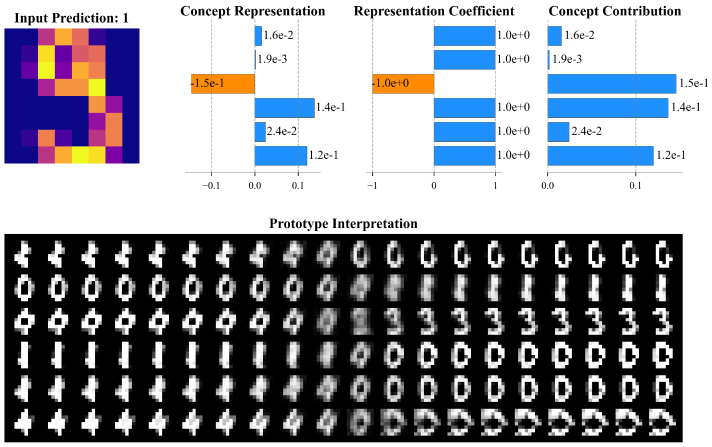
Prototype interpretation of an image from the validation set in Group 2 (beta = 4).

The Group 2 configuration, which exhibited the highest classification accuracy, was selected for an in-depth case study. As depicted in Figure 6, for a sample predicted as the digit 9, the concept representations for six concepts generated by the concept generator were plotted, along with the corresponding representation coefficients obtained through the feature extractor.

Given that continuous variation in the latent space of QVAE is meaningful, the operation of a concept dimension can be demonstrated by incrementally adjusting its value and decoding the reconstructed image. For instance, in this sample interpretation, the first concept represents a transition from the digit 4 to 6. The stronger the concept representation, the closer the sample resembles the digit 4. The second concept depicts the transformation of a zero gradually breaking into a 3, and then further reducing the lower semicircle to form a 2.

While encouraging concept diversity, some overlap between concepts was observed. This overlap arises mainly due to the complexity of the data and inherent correlations among features in the dataset. Although the β-VAE framework encourages disentanglement by adjusting the β parameter, achieving complete independence among latent variables can be challenging, especially with highly interdependent features such as handwritten digits. This overlap reduces the clarity of individual contributions, making it more difficult to attribute specific effects to individual concepts in the model’s decision-making process.

Additionally, certain concept interpretations remain challenging for human understanding. Some concepts may not correspond to easily interpretable transformations or features, which can hinder the user’s ability to understand and trust the model’s explanations. This suggests that when designing interpretable models, both the disentanglement and comprehensibility of the concepts should be considered to better facilitate human understanding of the model’s decision-making process.

The interpretability of quantum machine learning models is influenced by several factors, including the training extent of the concept generator, the number of layers in the QNN, and the dataloader used. Careful adjustment and optimization of these factors can significantly enhance model interpretability, thereby increasing its reliability in practical applications.

## 7. Discussion and Conclusions

The design of the Concept-Driven Quantum Neural Network (CD-QNN) model can be comprehensively understood through a two-tier hybrid framework. At the concept generator level, a quantum-classical hybrid model, specifically a QVAE, is employed. Additionally, when the concept generator is viewed as a quantum model, the feature extractor and feature integrator form another layer of a quantum-classical hybrid model at a higher level. This design philosophy harnesses the complementary strengths of quantum and classical computing, thereby aiming to enhance overall performance.

CD-QNN exhibits considerable flexibility and configurability, allowing it to adapt to various input data types and task scenarios by selecting suitable components and configurations to achieve optimal performance. This adaptability enables CD-QNN to meet diverse application requirements, providing a robust tool for future research on the integration of quantum and classical computing.

The CD-QNN model signifies a paradigm shift in QNN design by underscoring the importance of interpretability. CD-QNN integrates the potent representational capabilities of QNNs with the interpretability of self-explanatory models by mapping input data to a human-understandable concept space and making decisions based on these concepts. This methodology offers intuitive explanations of the model’s decision-making process, thereby enhancing the model’s transparency and credibility.

The collaborative mechanisms among the feature extractor, concept generator, and feature integrator are designed to augment and balance the model’s expressivity and interpretability. Emphasis is placed on concept disentanglement, with discussed methods aimed at effectively achieving the disentanglement and representation of concepts, thereby more effectively capturing key information in the data.

The loss function design underscores the significance of two-stage optimization in enhancing the effectiveness of explanations while balancing model prediction performance and interpretability. By considering classification loss, reconstruction loss, and the stability of explanations comprehensively, CD-QNN can maintain high predictive accuracy while offering clear and meaningful model explanations.

As quantum systems scale, maintaining interpretability becomes increasingly challenging. CD-QNN addresses this by focusing on extracting the most critical concepts and limiting the number of concept dimensions through dimensionality reduction techniques like QVAE. Additionally, CD-QNN employs hierarchical approaches for very high-dimensional systems, where higher-level concepts are derived from simpler ones. Visualization tools such as t-SNE and PCA further aid in making high-dimensional concept spaces interpretable. By integrating domain-specific knowledge, the model aligns its decisions with recognizable phenomena, ensuring that interpretability remains within human cognitive limits even as system complexity grows. Future work will explore these strategies in larger quantum systems to validate their effectiveness at scale.

As the demand for model interpretability continues to increase, CD-QNN and its derivative technologies are anticipated to play a pivotal role in future quantum artificial intelligence research and applications. By providing intuitive explanations and maintaining high predictive accuracy, CD-QNN offers crucial support for the development of more reliable and interpretable quantum intelligent systems.

## Figures and Tables

**Table 1:** Configuration of the concept generator in CD-QNN.

Parameter	Value
Number of qubits	6
Number of layers	10
Dataloader	Amplitude
Measurement operators	30
Training epochs	10
β	1

**Table 2:** Configurations of the ablation experiments for CD-QNN.

Index	Description
1	Baseline configuration
2	Increase β parameter in QVAE to 4
3	Reduce the number of measurements in the quantum concept generator to 6
4	Increase the number of layers in the quantum concept generator to 30
5	Without scaling layer
6	Use rotation gate dataloaders
7	Train concept generator simultaneously during feature extractor training
8	Increase the number of layers in the QNN to 30, and increase the number of training epochs to 30
9	Use rotation gate dataloaders and without scaling layer

## Data Availability

The data presented in this study are available on request from the corresponding author.
